# Promoting Social Creativity in Science Education With Digital Technology to Overcome Inequalities: A Scoping Review

**DOI:** 10.3389/fpsyg.2019.01474

**Published:** 2019-07-02

**Authors:** David Aguilar, Manoli Pifarre Turmo

**Affiliations:** Faculty of Education, Psychology and Social Work, University of Lleida, Lleida, Spain

**Keywords:** creativity, collaboration, technology, science, education

## Abstract

Enhancing creativity and developing technology skills in the classroom are the future of education and can turn out to be powerful tools to smooth out inequalities in class. This paper presents a systematic scoping review study of the literature focusing on cases of social creativity and digital technology embedded in science education. To this end, 23 empirical studies were selected from several databases—all in English and subjected to a blind peer-review process—to address the interconnectedness of key themes encapsulated in the following three research questions: (i) which digital technology roles support collaborative and creative processes in science education? (ii) which forms of technology and technological features support and organize the aforementioned creative processes? and (iii) what pedagogical principles guide the promotion of social creativity using technology in science education and involve all the students? Results show that technology can play different roles in promoting social creativity: (1) as a tutoring device that nurtures some key science creative processes; (2) as a tool that shapes students' creative thinking; and (3) as a medium that builds the supportive environment to perform collective creativity processes. In our project, these three roles were performed using a wide range of web 2.0 technologies (e.g., web-based environments, digital platforms, mobile technology) that both engaged all students in active and rich user experience for collective knowledge creation and equipped all learners with the necessary skills that would turn them into active, i.e., dynamic and resourceful, citizens in a swiftly changing world.

## Introduction

The switch to digital has prompted high-speed social and economic changes on a global scale. In this respect, recent EU reports endorse education's innovative capacity and encourage the development of twenty-first century skills or 4C's competencies (Wegerif, 2015) namely *Creativity, Collaboration, Criticality*, and *Caring*. In the same vein, Cheng (2010) confirms that educational reforms around the world have recently introduced creativity as a key educational target.

Recent research in education also highlights the social and collaborative dimension of creativity. Sawyer (2012), a leading creativity researcher, states that a creation process can be enhanced by collaboration and focus on the processes involved, named as “process approach.” It follows then that a creative process can be explained as a set of mental activities which people engage in when they are creating collaboratively. Consequently, compulsory education can become a crucial environment to promote the necessary creative mindset in all students to become active and creative thinkers. Those students, once furnished with a broad range of thinking and creative skills, would be empowered to overcome any possible inequality. Despite intensive discourse in this area, there are still barriers to overcome in terms of creativity and innovation in compulsory education (Cachia et al., 2010; Bocconi et al., 2012). This paper aims to fill this gap by reviewing existing research on promotion of social creativity in science education through technology-enhanced learning environments as a tool to overcome inequalities among compulsory education students. In particular, we report on a scoping review related to the aforementioned topic. A scoping review is a rigorous form of secondary research that involves collecting, assessing and summarizing available evidence (Arksey and O'Malley, 2005). In our study, we interpret such evidence and identify the most salient features of existing research (e.g., Kitchenham et al., 2015) that can provide an insight into further research on new pedagogies that embed creativity in science education. Researchers can use scoping reviews to study and clarify complex concepts and refine future research inquiries. Nowadays, scoping reviews are considered as a useful tool for reviewing educational research across different topics, especially those that are new and contemporary (e.g., Major et al., 2018).

Creativity is a multi-faceted concept that has been studied from different approaches and perspectives (e.g., Kampylis and Valtanen, 2010). One of these theories holds that creativity can be considered as “the ability to produce work that is both novel and appropriate” (Sternberg and Lubart, 1999, p. 3). Loveless (2003) highlights that this definition expresses four characteristics of creativity: a fashioning process, pursuing purpose, being novel, and judging value. Thus, creativity can be understood as the combination of different skills, knowledge, motivations, and attitudes that individuals use to evaluate a variety of input ideas in multiple perspectives and dimensions to create a new, valuable and original idea or product. In this way, Yager (2005) says that academic science programs should be considered important in the formation of a creative mind. According to him, some of the main human abilities in this domain are the following: visualizing-producing mental images, posing high-level problems and questions, making decisions, combining ideas in new ways, promoting critical thinking, solving problems, or designing devices and products that are novel and have a social or personal value.

On the other hand, there is another line of research that frames creativity on sociocultural theory and highlights the cultural and social settings where creativity is developed. In this respect, an increasing number of studies focuses on how creativity is developed inside a group or community and how organizations and groups can provide creative answers to new social and economic challenges. This socio-cultural conceptualization of creativity stresses the role of intersubjectivity, communication, collaboration, and dialogical interaction in the creative expression (Glăveanu, 2008, 2010). This novel line of research has coined such concepts as *social creativity, collaborative creativity, group creativity*, or *distributed creativity* (Sawyer, 2012; Glăveanu, 2014). The present paper focuses on this strand of research and advocates that creativity cannot be reduced to a set of psychological processes, studied in isolation from their social, material, and temporal context (Glăveanu, 2018). Therefore, in this scoping review we only take into consideration those studies that stimulate creativity in collaborative environments.

Recent research in the arena of computer-supported collaborative learning points out to the fact that technology provides a set of tools that can enrich the learning context and nurture social creativity processes (Lee and Chen, 2015; Henriksen et al., 2016).

In the context of educational research, technology has shown great potential in coordinating and orchestrating such creative processes as finding information, representing concepts, arguing, and sharing ideas, which lead to the promotion of collaborative and creative thinking (Gijlers et al., 2013). Online settings, mobile tools, digital blackboards are clear-cut and distinct examples of technologically-rich learning media. Such media can support successful teaching and learning practices while catalyzing skills such as creativity, cooperation, collaboration, or communication (Kumar and Sharma, 2017).

Curricular reforms around the world highlight the need to design technology-enhanced pedagogy for promoting collaborative creativity skills as key curricular objectives to be introduced in different subject domains or curriculum areas (Craft, 2012). Actually, *science* is a domain that can contribute to this end. The 2015 report of the European Commission on Science Education (http://ec.europa.eu/research/swafs/pdf/pub_science_education/KI-NA-26-893-EN-N.pdf) remarks on the relevance of the STEAM skills (i.e., *S*cience, *T*echnology, *E*ngineering, *A*rts, and *M*athematics) for solving current social challenges in creative and novel ways. In particular, science subjects are potentially creative social environments since they favor interaction of a series of factors including *domain-specific knowledge*, divergent thinking, imagination and visualization, and a social dimension (Hadzigeorgiou et al., 2012). These aspects can be developed through different pedagogical approaches that have been reported to enhance creativity in science classrooms, namely creative writing, inquiry-based or problem-based learning, and video gaming (Kind and Kind, 2007; Hadzigeorgiou et al., 2012; Wimmer, 2016). In particular, inquiry-based learning has been widely used to propel creativity in science education (Kind and Kind, 2007). When applying this pedagogical approach, students follow a set of steps, referred to as the *inquiry cycle*, which consists of orientation, conceptualizing (e.g., asking questions, generating hypothesis), investigation (e.g., exploration, experimentation, and data interpretation), conclusion and discussion (e.g., communication and reflection) (Barrow, 2010; Pedaste et al., 2015). Remarkably, this pedagogical approach is in line with the stages of the creative process suggested by Sawyer (2012).

### Scope and Aims of the Analysis

In the last decade, science education has progressively launched a whole raft of interactive and mobile technologies which are now extensively used both in scientific research and everyday activities. The introduction of interactive technology in science classrooms has opened up new possibilities and challenges in the design of learning scenarios that promote social creativity skills among all students. Some review studies analysing the development of creativity in science education can also be found in educational literature (Hadzigeorgiou et al., 2012). However, there is a lack of review studies that focus on the analysis of pedagogical variables that can be aligned with affordances of technology to support and orchestrate collaborative and creative processes in science education applicable to all students. This scoping review aims to fill this research gap and provide new and valuable pedagogical insights into designing technology-enhanced science projects that can offer support to the students' development of co-creativity processes and, by so doing, equip all students with key contemporary skills. Consequently, this pedagogical knowledge would contribute to reducing differences among students and increasing their opportunities to become active and creative thinkers. With this scoping review, our aim is to give an overview of works in this budding field. This work is not a systematic literature review or meta-analysis. Systematic reviews tackle precise questions, with defined methodologies to evaluate and discuss study quality (O'Brien et al., 2010). In a scoping review, the purpose is to examine and summarize a range of evidence, presenting board findings and relationships to convey the breadth, depth, and innovation of the aforementioned field of study (Arksey and O'Malley, 2005; Levac et al., 2010). Thus, the present study establishes connections between creativity, technology, and collaboration in science education that can support further research and research analysis with different intentions and designs (Levac et al., 2010; Kitchenham et al., 2015).

In our study, we focus on research carried out on elementary and secondary education students (compulsory education: 6–18 years old) as well as science teacher education. This study covers recent research carried out in the last 10 years, a decade heavily marked by higher integration of web 2.0 technologies in science classrooms. The whole Web 2.0 concept was first coined in 2004 by O'Reilly (2004) in reference to a group of technologies (e.g., blogs, wikis, web-based environments, etc.) that promoted collaboration and the exchange of information between users. But, according to Scopus, it was not until 2008 when the number of studies that showed the incorporation of this kind of technologies in science education expanded, which justifies the interest of focusing the present study in the last decade. Specifically, we will address the following research questions (RQ):

RQ 1. Which specific roles of digital technologies have been identified in the existing literature that support collaborative and creative processes in science education?
RQ 2. Which forms of technology and technological features have been used to support and orchestrate collaborative and creative processes in science education?
RQ 3. What pedagogical principles have been identified focusing on the promotion of social creativity using technology in science education practices and for all the students?

## Methodology

### Literature Search and Criteria for Paper Selection

A scoping review was carried out following the methodological framework initially proposed by Arksey and O'Malley (2005) and improved years later by Levac et al. (2010). This framework establishes five different stages: (a) identifying the corresponding research questions; (b) identifying studies that are relevant for those questions; (c) selecting studies; (d) charting data; and (e) summarizing and reporting results. Automated searches of selected digital libraries were carried out to identify the most relevant studies on the development of social creativity skills using digital technology in science education. We selected the main articles within this field from Scopus and Web of Science (WOS), which are relevant databases for educational research.

Firstly, we examined the titles, abstracts, and keywords of different studies previously found as relevant (Jang, 2009; Seitamaa-Hakkarainen et al., 2010; Sullivan, 2011). In this first stage of the search method, we identified the following set of keywords: “science,” “creativity,” “learning,” “technology,” “computer,” “collaboration,” and “collaborative.” Then, some keywords were iteratively developed after examining the titles, abstracts and keywords of studies identified in the first search stage. Thus, the following keywords were also included in the search process: “mobile,” “computer,” “laptop,” “robotic,” “virtual,” “web,” “wiki,” “online.” Finally, the following search string was created to cover the variables proposed in the research questions (science, collaboration, creativity, and technology):

“science” AND “creativ^*^” AND “collab^*^” AND “learn^*^” AND (“techno^*^” OR “mobile”, “computer” OR “laptop” OR “robotic” OR “virtual” OR “web” OR “wiki” OR “online”).

### Inclusion and Exclusion Criteria

The authors of the present paper conducted all the screening stages and discrepancies and citations that partially meet the criteria were solved by consensus since member checking is a well-established procedure to build up “trustworthiness” in qualitative research (Toma, 2006, p. 412).

Table 1 presents the general inclusion and exclusion criteria applied that ensure that only relevant literature for the objectives of the present work were accepted.

**Table 1:** Inclusion and exclusion criteria.

**Inclusion criteria**	**Exclusion criteria**
- Publications were included if they report on the development of social creativity or a creative process with technology. - Studies that are related to the domain of experimental science. - Publications that were peer-reviewed. - Studies focused on elementary and secondary education students as well as science teacher education students. - Papers published between 2008 and 2018.	- Conference proceedings were excluded as we focused on completely blind peer-reviewed texts. - Books and book chapters were discarded because of accessibility difficulties. - Publications not focused on the domain of experimental science (e.g., we excluded studies focusing on mathematics or social sciences). - Publications that are not focused on the targeted educational levels. - Studies that were not written in English.

**Figure 1:**
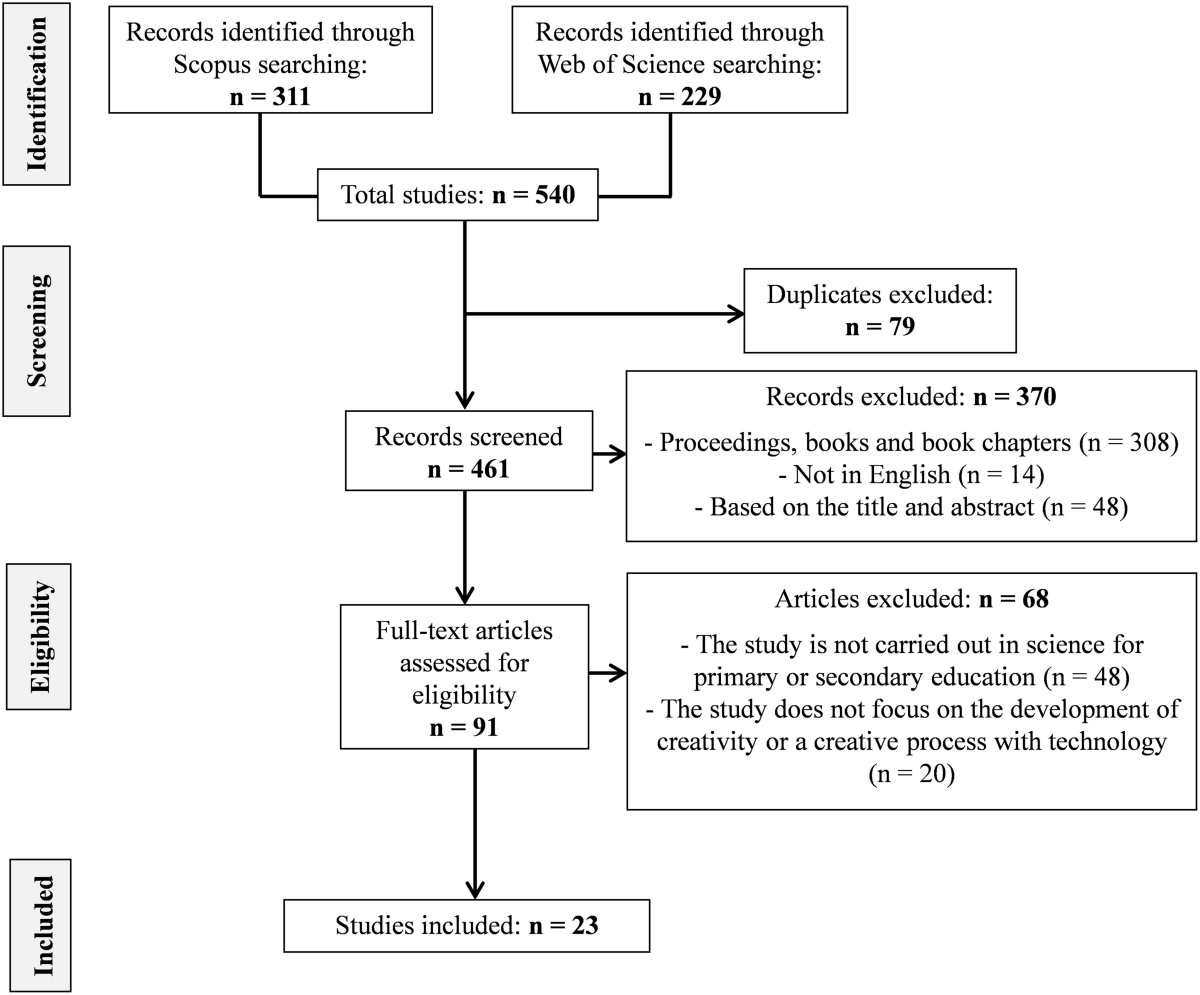
Selection process of the studies for our review.

More specifically, Figure 1 summarizes the screening procedure followed where the aforementioned inclusion and exclusion criteria were applied in order to select the key studies. The initial selection comprised 540 articles and, after excluding duplicated texts, was shortlisted to 461 articles. During a first screening, we excluded conference proceedings, books, books chapters, and papers whose title and abstract were unrelated to the purposes of the present review. After this initial screening, 91 full-text papers, considered potentially relevant for this study, were fully read and assessed on their adequacy to our research purposes. As a result, 48 of them were discarded since they were not related to the domain of science (excluding mathematics or social sciences studies) or not aimed to our targeted educational levels. Also, 20 more articles were discarded because they did not explicitly report on the development of a creative process in the domain of science using technology. This process resulted in a final selection of 23 relevant documents for our research.

### Data Extraction, Analysis, and Synthesis

In order to answer the proposed research questions, the following five ranges of data values were extracted from the 23 selected studies: (a) study aims and objectives; (b) participants (age, level of education); (c) role and type of digital technology; (d) main pedagogical principles to promote creativity in science education for all students; and (e) collaborative learning pedagogies.

In order to address the research questions, data were extracted from the abstract, findings, or conclusions sections of the studies. The authors of the present paper participated in the process and the data obtained were stored in evidence tables. This facilitated the handling of the information extracted including charting of key features of the selected studies. To develop a critique and identify key themes, a narrative synthetic approach was applied (Davis et al., 2009). In this phase, we analyzed and categorized separately the data and, later, discussed and agreed collaboratively a set of themes deriving from the initial research questions.

### Methodological Limitations

The search only considered peer-reviewed papers written in English between 2008 and 2018. Admittedly, the choice of keywords used or omitted and the data-bases used may have limited our findings. Thus, other studies relevant to the topic of the present work may have been excluded. It is also possible that methodological decisions for extracting and synthesizing data could have introduced some subjectivity. Some actions to mitigate the impact of some of these limitations could be to examine the reference list of the selected publications and perform trial searches.

## Results

Firstly, this section provides a brief overview of key findings of the scoping review. Secondly, it lays out the results that tackle the three research questions posed in this review.

Table 2 provides an overview of the core data extracted from the selected studies aiming to give an answer to the proposed research questions.

**Table 2:** Summary of the reviewed studies that report on creative practices with digital technologies in the science domain.

**References**	**Grade**	**Role of technology**	**Form of technology**	**Pedagogy**		
				**Science content**	**Student centered activity**	**Teachers' role**
Plessis and Webb, 2008	Elementary education (12 years old)	Medium	Web-based technology (Encarta) and multimedia presentations	Different real-life science topics (marine life, birds, reptiles)	Construct a creative knowledge-object about a science topic and produce a visual presentation	Give specific and planned guidance
Jang, 2009	Secondary education (13 years old)	Medium	Web-based technology	Different real-life science topics (e.g., evolution, nutrition, buoyancy, biodiversity)	Research about a scientific real-life phenomenon and produce a visual presentation	Give specific and planned guidance
Smith et al., 2009	Secondary education (13–16 years old)	Medium	Handheld sensors (carbon monoxide sensor), GPS, note tracker, blogs	Contamination around the school	Research about a scientific real-life phenomenon	Give specific and planned guidance
Arnold et al., 2009	Pre-service teachers	Tutor	Instructional movies and online discussion forum	Life and the environment (e.g., ecosystems, structure and functions of plants, animal life)	Construct a creative knowledge-object about a science topic	Support the dialogue of students
Seitamaa-Hakkarainen et al., 2010	Elementary education (10–12 years old)	Medium	Knowledge forum	Study the properties and design of an artifact (e.g., lamp, properties of light)	Construct or design of a new product	Organizer of the shared knowledge practices
Wishart and Triggs, 2010	Elementary and secondary education (11–18 years old)	Medium	Mobile phones and Evolution (authoring tool)	Different real-life science topics (e.g., plants, soils, minerals)	Research about a scientific real-life phenomenon and produce a visual presentation	Give specific and planned guidance
Sullivan, 2011	Elementary education (12 years old)	Tool	Robotics	Light-sensor-enabled robotics problem	Construct or design of a new product	Support the dialogue of students
Zhang and Sun, 2011	Elementary education (9–10 years old)	Tutor	Knowledge forum	Light	Construct a creative knowledge-object about a science topic	Organizer of the shared knowledge practices
Hong et al., 2013	Secondary education (13–14 years old)	Tool	Robotics	Design and program a wooden robot	Construct or design of a new product	Give specific and planned guidance and support the dialogue of students
Lee et al., 2013	Elementary education (7–12 years old)	Medium	Tablet-PCs	Different real-life science topics (e.g., materials, plants, minerals)	Research about a scientific real-life phenomenon	Give specific and planned guidance
Yang and Chang, 2013	Secondary education (13–14 years old)	Tool	Design digital games	Biology	Construct or design of a new product	–
Hemling et al., 2014	Secondary education	Medium	Web-based environment	Microfluidics and properties of acid-base chemistry	Construct or design of a new product	Enrich and structure student's collaborative inquiry
Lee et al., 2014	Elementary school teachers	Tool	Robotics and online spaces (i.e., Moodle)	Lego® Educational Toolkits for constructing physical artifacts (e.g., a mountain stretcher)	Construct or design of a new product	Give specific and planned guidance and support the dialogue of students
Chen et al., 2015	Pre-service teachers	Tool	Wikis	Life and understandable science content (e.g., Newton's law, Bernoulli's law)	Construct a creative knowledge-object about a science topic	Give specific and planned guidance
Kim et al., 2015	Elementary education (10–11 years old)	Medium	Mobile phones	Air, wind, force, and energy	Construct or design of a new product	Give specific and planned guidance
Mudaly et al., 2015	Pre-service teachers (20–24 years old)	Tool	Digital animation and digital concept-mapping	Socially relevant science topics: health issues (e.g., HIV, weight, malnutrition), environmental issues (e.g., pollution, climate change)	Construct a creative knowledge-object about a science topic	Organizer of the shared knowledge practices
Ramírez-Benavides and Guerrero, 2015	Elementary education (4–6 years old)	Tool	Robotics	Programming in mobile devices for Lego Mindstorm (abstract and logic thinking)	Construct or design of a new product	–
Guo and Woulfin, 2016	Elementary and secondary education (6–18 years old)	–	Web-based environment, wikis	Different real-life science topics (e.g., gardening project, decomposition of natural and human-designed materials)	Research about a scientific real-life phenomenon	Give specific and planned guidance
Lin et al., 2016	Secondary education (14–15 years old)	Tool	Google Docs and digital concept-mapping	Design of a water rocket with a hydrogen-oxygen engine	Construct a creative knowledge-object about a science topic	Give specific and planned guidance
Kumar and Sharma, 2017	Elementary and secondary education (6–18 years old)	Medium	Cloud technology (e.g., virtual laboratories)	Different real-life science topics (no specific examples are given)	Research about a scientific real-life phenomenon	Organizer of the shared knowledge practices
Poce et al., 2017	Pre-service teachers	Tutor	Orbis Dictus (digital platform for online education)	Marine biodiversity	Construct a creative knowledge-object about a science topic	Give specific and planned guidance
Ridwan et al., 2017	Secondary education (15–17 years old)	Medium	Handheld sensor (pH meter) and mobile phones	Chemistry concepts (solubility, acid base, petroleum, hydrocarbon)	Research about a scientific real-life phenomenon	Give specific and planned guidance
Sanabria and Arámburo-Lizárraga, 2017	Secondary education (17–18 years old)	Medium	Interactive application that supports Augmented Reality	Digital creation of *learning objects* in a STEAM context	Construct or design of a new product	–

A first overview of Table 2 shows that the proportion of research performed in elementary (students between 5 and 12 years old) and secondary (students between 12 and 18 years old) education is practically even (*n* = 10 for elementary education and *n* = 11 for secondary education). In three of these works, the study was undertaken with a sample that includes both elementary and secondary students. On the other hand, only five studies were carried out with either pre-service teachers or in-service elementary or secondary teachers.

**Figure 2:**
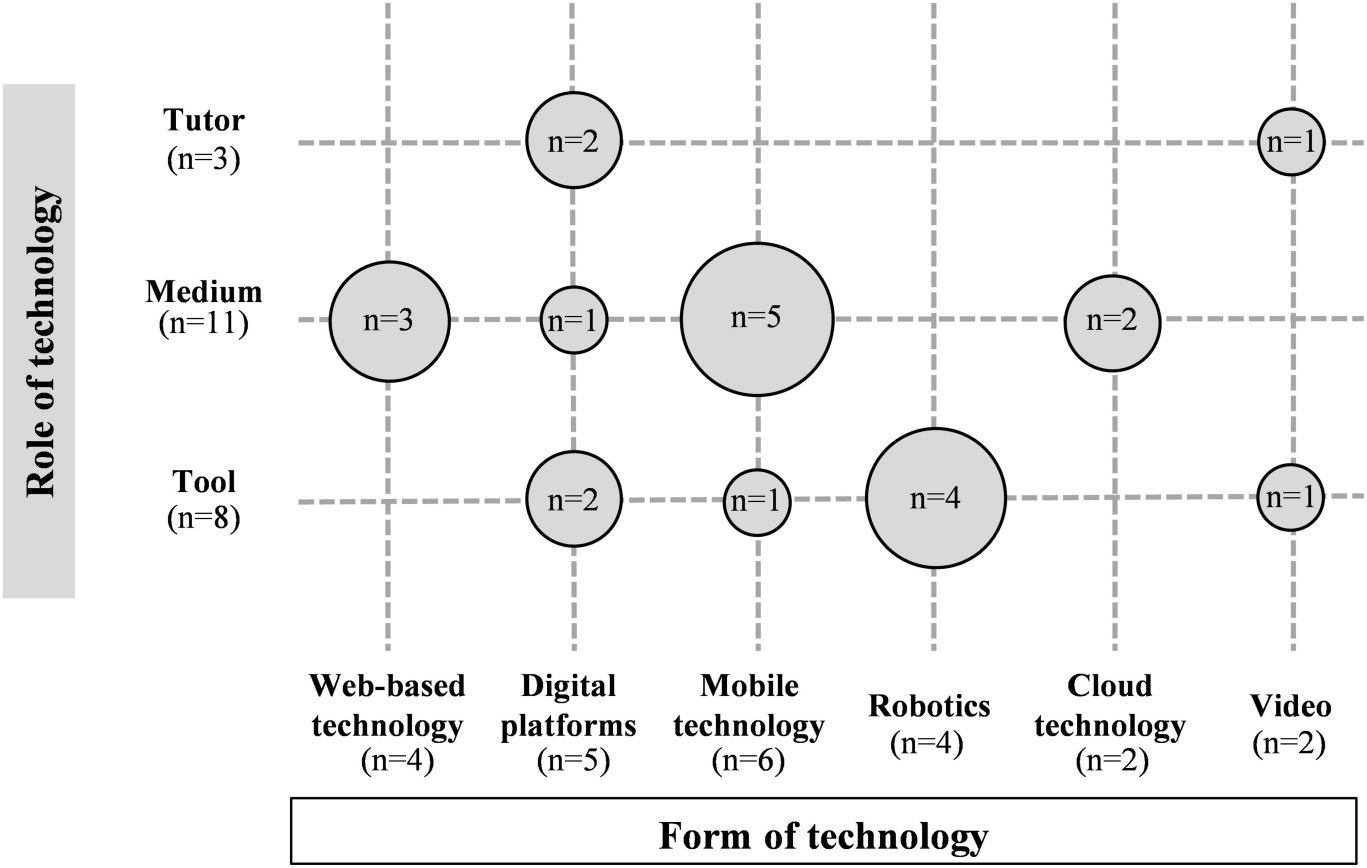
Roles of technology reported by the studies reviewed according to the specific technology used.

Figure 2 displays the results in relation to the roles that technology plays in fostering students' social creativity in science education in the reviewed studies (RQ1) and in relation to the forms of technology used to support social creativity (RQ2).

In relation to which *specific roles of digital technologies could be identified in the existing literature to support collaborative and creative processes in science education* (RQ1), the qualitative analysis of the papers selected for this review identified three different roles of technology in fostering students' social creativity in science education: (1) technology as a tutor that supports and facilitates the emergence of key creative processes in science; (2) technology as a tool which utilization and appropriation of its characteristics by the students becomes an instrument to think creatively; and (3) technology as a medium or an environment that stimulates collaborative and creative thinking. These three roles are not mutually exclusive, e.g., a robotics, can be used in initial stages of the educational project as a tutor to script and guide the student's learning of specific design processes and, in more advanced stages, this technology can be used as a tool for thinking creatively in order for students to use the programming language autonomously.

As shown in Figure 2, technology as a medium and technology as a tool are the most frequent roles among the studies reviewed (*n* = 11 and *n* = 8, respectively). Both roles were introduced through the use of a wide range of digital technologies.

Six different forms of digital technology were identified in the studies reviewed in order to promote social creativity in science education for all students (RQ2). The studies analyzed used mainly web-based environments (*n* = 4), digital platforms (*n* = 5), mobile technology (*n* = 6), and robotics (*n* = 4). A limited number of studies introduced cloud technology (*n* = 2) or video (*n* = 2). In particular, when technology was introduced as a medium for promoting social creativity, web-based (*n* = 3) and mobile (*n* = 5) technologies were the most frequent ones. When digital technologies were implemented as a tool, then, robotics (*n* = 4) was the main one. Technologies with a tutoring role were introduced to a much lesser extent (*n* = 3), being video and digital platforms the only digital technologies implemented with the aforementioned role.

**Figure 3:**
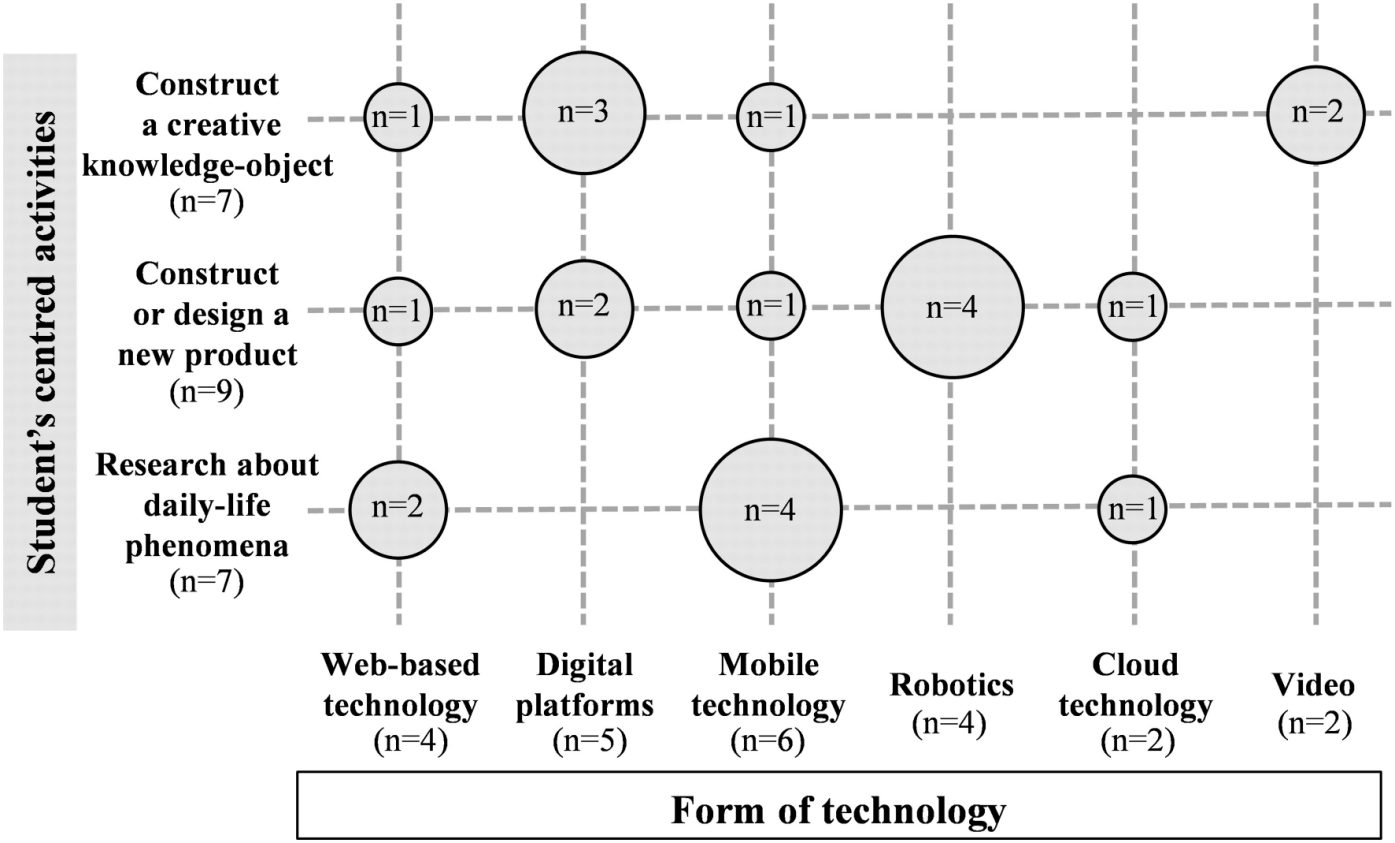
Type of student-centered activities reported by studies selected according to the form of technology used.

Furthermore, the studies also reported on different pedagogical principles and scenarios where knowledge creation was performed (RQ3). On the one hand, authentic science problems were tackled in the selected studies in which three different types of student-centered activities were designed to promote skills and knowledge for solving scientific problems: i.e., construct a creative knowledge-object (*n* = 7), construct or design a new product (*n* = 9), and research about daily-life phenomena to facilitate knowledge building (*n* = 7). Different forms of technology were used to perform these three different types of student-centered activities, as Figure 3 shows.

As seen in Figure 3, web-based technology, digital platforms, mobile technology and robotics are the forms of technology most frequently used to mediate in solving the three types of student-centered activities. Furthermore, it is worth emphasizing the overriding role of robotics to enhance learners to construct or design a new product (*n* = 4), and mobile technology when students are asked to do some research on a daily-life phenomenon (*n* = 4).

Next, we address the discussion of the results obtained in relation to the three research questions proposed in this review.

## Discussion

### Roles of Technology in Fostering Students' Social Creativity in Science Education (RQ1)

Knowledge in the twenty-first century is highly shaped by the development and affordances of technology (Higgins, 2014). Certainly, a specific technology imposes certain constrains and opens up a range of opportunities available to a group of learners. In this line, Wegerif (2015) claims that technology shapes thinking from within because it impacts on how we actually think and interact with others.

Although technological settings establish preconditions for educational opportunities, they do not causally determine these activities, or their peer-assisted learning outcomes (Oliver, 2011). Because of this, there is a need to study how collaborative activities occur interactively in a rich-technology context in order to further understand the students' peer-assisted learning results.

Following this argument, in this review paper we analyse how technology is used to promote social creativity in science classes. Thus, we study how interaction between participant agents (i.e., teachers, students, and community) and technology affordances are mutually shaped to promote learning. This analysis will contribute to creating a more insightful discourse and conceptualizing the relationship between different technologies, the way in which they are used and the impact they may have on the users' creative thinking in science education.

The qualitative analysis of the papers selected for this review identified three different roles of technology in fostering students' social creativity in science education: (1) technology as a tutor; (2) technology as a tool; and (3) technology as a medium for collaborative and creative thinking (see Figure 2). As mentioned above, these three roles are not mutually exclusive and a particular technology can be used for different purposes or could even be used for different educational objectives during the different students' learning stages. In the following sections, we discuss how technology is used in these three different roles in science classrooms.

1. **Technology as a tutor of creative thinking**. Digital technologies can be seen as tools available to facilitate key creative processes in science. In this situation, technology contributes to increasing opportunities to facilitate creative processes in science. The teacher plays an important role in using technology because s/he designs and supervises activities requiring the use of technology.
  In these activities, specific guidance is given by means of scripts or prompts that stimulate the performance of a specific creative process. For example, technology is used to tutor the understanding of the typical characteristics of the language of science and create the structure of a creative text with a scientific topic through cooperative writing (Poce et al., 2017). Technology also tutors the development of professional knowledge in an activity in which pre-service teachers design instructional movies following a script while participating in online guided discussion activities (Arnold et al., 2009). The educational activity is usually monitored by an e-tutor who provides information on the use of the platform and engages students' participation to solve a creative activity (Poce et al., 2017).
  The relationship between technology and learning underlying the use of technology as tutor is that technologies possess inherent qualities, and are capable of having a particular “impact” and/or “effect” on learners if used in a *correct* manner in a science classroom. The “correct” usage of technology is usually scripted by the teacher, who decides which technology is used, how it is embedded in a specific science methodology and when it is used.
2. **Technology as a tool for thinking creatively**. The socio-cultural theory argues that individuals learn to think through internalizing the use of cultural tools—such as language or technology—which, in turn, become cognitive tools or tools to think with (Vygotsky, 1987). This “instrumental genesis” (Rabardel and Bourmaud, 2003) addresses the connection of human agents and technical artifacts through the concept of instrument. An instrument is a heterogeneous entity, composed of both a technical artifact and a human agent. The instrument emerges from a “double-development movement” which connects the artifact and its utilization scheme, while agents adapt and give form to the artifact (Overdijk et al., 2012). Technology can be seen as an “instrument” of sorts, inasmuch as it is a tool used to shape and develop a creative activity. Therefore, instrumentalization changes both the tool and its user.
  In science education, several studies (Sullivan, 2011; Hong et al., 2013; Lee et al., 2014; Ramírez-Benavides and Guerrero, 2015) have delved into instrumentalization processes where tools such as robotics serve students solve problems creatively and shape the way they think. Usually, these tools are introduced in science activities whose solution involves solving a challenge by creating a product through technology. For instance, Sullivan (2011) explores the development of a creative solution designed by students working collaboratively to solve a light-sensor-enabled robotic problem in a sixth-grade science classroom. In this study, students solve creatively a challenge using robotic tools while they plan, share opinions, and build or integrate ideas from other people. Hong et al. (2013) discuss the importance of an after-school Science-Technology club in which junior high-school students engage themselves in solving a scientific problem, in particular, the creation of a robot to win a nationwide contest. Through these proposals, students develop informal science, think about creative ways to complete their project and search new ways of integrating their knowledge and experience in science and technology to, eventually, assemble a robot.
  In the same line, Mudaly et al. (2015) and Lin et al. (2016) study how a structured concept-mapping activity supported by digital technologies and social media might be used creatively for co-constructing science knowledge through observation, modification, and interaction.
  Finally, in all of these studies, the process of thinking creatively with a dedicated technology—such as robotics or concept-mapping—converts the technology into an instrument of thinking that promotes the development of key creative processes to complete their project such as: idea and knowledge creation, development and improvement, idea selection or idea representation, as well as elaborate high-quality scientific knowledge.
3. **Technology as a medium for social creative thinking**. Digital technologies offer many possibilities which can be exploited and experimented to support the performance and orchestration of creative processes. In this respect, digital technologies can contribute to establishing further creative processes, by providing new tools, media and environments for learning to be creative and learning through creativity. Learners and teachers can use different technologies to design an educational environment which stimulates, orchestrates and supports specific creative processes such as developing ideas, making connections, fostering collaborations, and encouraging imaginative expressions.

In the selected studies, technology has been used preferably as a medium for social creative thinking (see Figure 2). In particular, the main roles of technology as a medium in the development of creative experiences in inquiry-based science found in this literature review are the following three:

- *Cultivate specific creative processes by providing a wide range of technologies*. Plessis and Webb (2008) and Jang (2009) designed a web-based environment that promotes students' abilities to discover, think, and discuss problems by posing key real-life problems, providing key resources, enhancing discussion forums, answering online questions and offering links to related and useful websites. Sanabria and Arámburo-Lizárraga (2017), as members of a STEAM project, implemented a cognitive-pedagogical approach using Augmented Reality (AR) which successfully nurtured the development of key creative processes. The project guided the students through three modules in order to fulfill the creative process. Module I familiarized teams of learners with a specific topic and the development of six key creative processes: observation, combination, association, grouping, discernment, and evaluation. Module II revolved around digital creation and development of creative design processes as: generation, modification, and visualization. Finally, Module III focused on displaying and communicating.
  These studies reveal the importance of integrating network and inquiry activities into a real-life science experience. The situations that participants encounter along the process can stimulate creative thinking, flexibility, originality, and elaboration of fluency to express scientific ideas.
- *Orchestrate the social creativity process in science education*. Kim et al. (2015) proposed a rich technology-enhanced project in which 5th grade students designed their own experiments with mobile phones and relevant applications and sensors. The project, which encouraged students to engage in problem-solving by finding solution designs, orchestrated, and scaffolded the following scientific processes: (re)defining the problem, planning action, implementing, evaluating, and specifying findings, documentation, evaluation, and finally, reporting scientific phenomena.
  A similar study carried out by Seitamaa-Hakkarainen et al. (2010) aimed to promote genuine inquiry at primary education level by analyzing and designing artifacts within a cultural context. The study engaged students in a collaborative inquiry using the virtual platform Knowledge Forum which scaffolded and orchestrated the different stages and processes of the collaborative and creative design: namely, defining a task and its constraints, creating conceptual, and visual design ideas, evaluating design ideas and constraints, connecting to an expert culture and facilitating data collection, experimentation, and evaluation.
- *Expand the creative learning space*. Mobile technologies can exemplify this role since they provide a diverse and rich educational context beyond the classroom (Plessis and Webb, 2008; Smith et al., 2009; Wishart and Triggs, 2010; Lee et al., 2013; Kim et al., 2015). The provision of such new learning spaces can facilitate the students' improvement by developing new ideas, making connections between concepts and collaborating with other participants. For example, Lee et al. (2013) report on the educational effects of mobile-technology-based science classes on the performance of creative activity-oriented discovery processes. The combination of mobile technology with social networking services for carrying out inquiries facilitated the understanding of scientific knowledge and propelled the students' interest and motivation.

### The Forms of Technology and Features That Have Been Used to Support and Orchestrate Collaborative and Creative Processes in Science Education (RQ 2)

Different forms of technology have been introduced in the studies analyzed and all of them offer a wide range of specific properties that support creativity processes in science. Web 2.0 technology activities that promoted social creativity were grouped together into different categories.

The vast majority of studies included in the present study (*n* = 6, Figure 2) incorporated **mobile technologies** (Figure 2). This form of technology is being gradually integrated into education on account of its capacity to expand the learning space beyond the classroom and enrich the learning contexts. Kim et al. (2015) claim that a wide range of mobile platforms can help students better observe the world, record significant moments, synthesize their ideas, and increase their engagement in science. Among the selected articles, different mobile technologies stand out for supporting collaborative and creative processes in science. For instance, *Tablet PCs* with online connectivity are used to support scientific discoveries with flexibility of time and space. Lee et al. (2013) describe how students used these devices to access scientific content wherever and whenever they needed, and collect real data and evidence (e.g., photos, videos, sounds) for their scientific inquiries. *Mobile phones* are also highlighted in a project by Kim et al. (2015) with a special focus on two main features: (i) they can provide instant communication between peers; (ii) they can be used for gathering real-time data (e.g., angles, time, or distances) by using specific applications. Wishart and Triggs (2010) also emphasize that mobile phones expand the learning spaces beyond the traditional formal environments and allow taking photographs and notes during a visit to a museum. Finally, *Scientific handheld sensors* are also highlighted as professional instruments to perform specific field measurements. In particular, Smith et al. (2009) report on the use of a handheld carbon monoxide sensor to monitor air pollution in the school surroundings while Ridwan et al. (2017) depict how students tracked the acidity of water from an aquarium using a pH-meter.

Five studies report on the use of different specific **digital platforms** for managing data, information, knowledge and supporting a collaborative creative process in science education. In particular, two of these studies highlight *Knowledge Forum* as a collaborative online platform rooted into research on knowledge building discourse. The platform provides a shared and multimedia space where students' ideas are given a visual and public representation: they can share information, participate in online debates, reflect upon their findings, launch collaborative inquiries and build new ideas together. Seitamaa-Hakkarainen et al. (2010) also used Knowledge Forum affordances to orchestrate learner's inquiry practices for designing new artifacts. Likewise, Zhang and Sun (2011) used this platform as a space for supporting reading practices in science where students could contribute with their own ideas, examine their peers', as well as revise, combine, synthesize, and build up new ideas. In both studies, Knowledge Forum provided an on-line space of permanent dialogue and streamlined insights through sustained knowledge-building discourse and management of group flow. These studies emphasize the significance of organizing collaborative inquiry processes in a visual way by using the multimedia facilities of this digital platform. These facilities provide students with new opportunities to better identify, represent, and structure the different skills and processes implemented along an inquiry activity which, according to scholars, is one of the main hurdles students encounter when using digital platforms (Piekny and Maehler, 2013).

*Google Docs* is another digital platform reported in the studies. This platform is a web-based collaborative word processor that enables discussion and interaction in order to build, co-edit, and share information. Lin et al. (2016) emphasize the effectiveness of Google Docs for performing creative concept maps about physics. In this line, Wishart and Triggs (2010) introduce one digital platform called *Evolution*, a multimedia collaborative authoring tool for creating interactive presentations on scientific findings in a museum. This platform followed the principle of “learning by teaching” and provided all the necessary functionality and templates for organizing the students' ideas and preparing communication with their peers. Likewise, Poce et al. (2017) focus on *Orbis Dictus*, an online multilingual educational environment that offers a flexible and dynamic setting with the necessary tools for scaffolding a didactic path. In this study, this platform is introduced for creative co-writing activities in science. It allows organizing a set of on-line group activities for students to reflect on the levels of complexity and language used in a series of texts and, subsequently, participating in co-writing creative scientific texts. *Wiki* is the last digital platform reported in the studies analyzed. It is a collaborative web page authoring system where different users can freely contribute, create, or modify any content, even contents previously created by other users. It allows uploading multimedia information such as texts, videos, images, hyperlinks, or documents (Kim et al., 2012). Chen et al. (2015) explore the influence of wiki environments on the growth of pre-science teachers in a *T*echnological, *P*edagogical and *C*ontent *K*nowledge (TPACK) framework. Wiki facilitates storage, presentation, and modification of ideas, organization of teaching materials, instructional plans as well as the members' collaboration to come to an agreement on a series of creative instructional approaches related to different science topics.

**Web-based environments** were highlighted in four of the studies analyzed. This form of technology focuses on the World Wide Web as a tool to obtain, receive and manipulate up-to-date information in different areas through a single computer (Guo and Woulfin, 2016). Jang (2009) and Hemling et al. (2014) describe the application of web-site environments to decrease the complexity of learning scientific goals by making the task structure explicit, motivating and able to guide students through the inquiry process. By means of a web-site environment, the teacher provides explicit instructions, online conceptual presentations, task structuring scaffolds and experimentation hints. Students, in turn, become interactive learners, collaborate between them and enrich their problem strategies to solve creatively a science problem.

Four of the studies analyzed highlight **robotic technology** as it provides new opportunities for introducing students into open-ended, goal-oriented tasks. These tools allow students to interplay between logic and reasoning and between play and seriousness (Sullivan, 2011). In robotic problem-solving activities, students construct digital artifacts by following a programming cycle that consists of: (i) writing and testing a program; (ii) diagnosing problems with the program or structure of the device; (iii) proposing and arguing changes to the program or structure; (iv) making changes to the program and testing the device again. The studies developed by Sullivan (2011), Hong et al. (2013), Lee et al. (2014), and Ramírez-Benavides and Guerrero (2015) emphasize the benefits of following an inquiry cycle using robotic technology. They are the following four: (a) it provides students with new opportunities to research ideas and to experiment; (b) students can move between rule acquisition and rule modification because risks can be taken safely as it is possible to make and remake, repurpose, and recycle easily and effectively; (c) it facilitates direct and swift application of choices and, by so doing, students can modify and improve their ideas collaboratively, and (d) it increases the students' motivation and playfulness, which fuels their creativity.

The introduction of **cloud computing technologies** is breaking new ground in education. These technologies facilitate innovation, creativity, and experimentation in science because they contribute to reducing costly infrastructures such as labs or scientific instruments. Kumar and Sharma (2017) describe the application of this technology to provide *virtual laboratories* or *virtual simulators* that emulate real environment technologies. These settings are safe and can be accessed anytime, anywhere and help students to understand concepts from real world contexts. In the same line, Sanabria and Arámburo-Lizárraga (2017) used the affordances of a particular cloud computing technology, *Augmented Reality (AR)*, to integrate 3D digital elements into the real world, provide real-time interaction and enrich the perceived information. AR allowed introducing abstract, difficult-to-assimilate and conventionally-inaccessible information which enhanced digital creation, organization, communication, management of information, and problem-solving skills.

Finally, **video** is reported in **two** of the studies analyzed and has been generally applied in science education as a representational and communicative tool. Mudaly et al. (2015) explore the use of video and digital animations as a medium for communication of a socially relevant science issue (e.g., health diseases). The authors remark that this tool provides unique opportunities to capture and present scientific contents while promoting critical thinking. On the other hand, Arnold et al. (2009) claim that producing a video can foster creativity since it allows students to record, organize and develop the ideas they want to share.

To sum up, the studies reviewed have used the affordances of a wide range of interactive Web 2.0 tools to promote the students' development of creative thinking skills. Also, such tools provide online and collaborative environments in which both teachers and learners can have a rich user experience and contribute with new and valuable knowledge to the online learning community.

### Aligning Pedagogy and Technology for Promoting Social Creativity in Science Education (RQ 3)

The selected papers followed a common approach toward learning that Paavola and Hakkarainen (2005) defined as the *knowledge creation approach (KCA)*. This approach claims that learning is not only a process of acquisition and construction of existing knowledge but it is mainly an active and contextualized process of constructing and creating new knowledge. Knowledge is created on the basis of personal and inter-personal experiences, in which all students' contributions are important. In addition, knowledge is created in activities developed in social contexts and by using and developing common objects of activity such as conceptual artifacts, practices, or products.

The selected papers designed different pedagogical scenarios to implement the knowledge creation approach for school learning purposes. The pedagogical scenarios are the following ones:

1. **Authentic science problems and science contents**. The selected papers emphasized the social character of knowledge by engaging students in solving authentic, real-life and complex problems that could arise inside or outside the educational institution. Rather than addressing mere pre-structured problems, fragmented according to subject-domain, students carry out inquiries by themselves and are engaged in real research related to science topics. The studies analyzed concluded that science problems engaged all the students around meaningful science in new ways and supported the development of new forms of scientific inquiry skills. Authentic learning is a key pedagogy to engage all students in a research-grounded scenario capable of promoting creative skills, regardless of their previous knowledge and therefore, contributes to reducing their initial differences. For instance, Kim et al. (2015) describe how a diverse group of students succeed in a problem-solving project where they performed iterative experimental designs to provide solutions and explanations to different scientific phenomena. One key aspect for the success of this project was the high amount of contributions from each and every participant involved.
2. **Extending the learning community and breaking boundaries between spaces and communities**. In this pedagogical approach, the classroom walls become more permeable to students' outside experiences and the classroom becomes a node, or “an intersection” (Leander et al., 2010 p. 336) within a background of different learning experiences. These learning backgrounds as grounded on wider social groups and on students' participation in life-long learning processes and practices can afford to effectively deal with societal challenges and with the contributions of all the participants. Indeed, the articles selected promote social interactions within different cultural settings. In these studies, different pedagogical strategies are used to promote the social character of knowledge. The strategies are the following:
  - *Students solve problems related to everyday science contents*: for instance, marine biodiversity (Poce et al., 2017), light and heat energy (Sullivan, 2011), forces and energy (Kim et al., 2015), reproduction and genetics (Yang and Chang, 2013), exploration of evolution or energy of nutrition (Jang, 2009). The process of solving problems of real-life science enables students to bring into class discussions of their own experiences as well as scientific knowledge and, thus, facilitates knowledge-construction processes.
  - *Students solve different kinds of information*. The use of global e-science data and different kind of facilities enables students to go through the experience of working as “real scientists” by developing skills related to data access, data visualization, and global scientific collaboration (Smith et al., 2009).
  - *Students expand the learning scenarios beyond the classroom and with experts other than teachers*. The idea is to expand the learning community and help students create new knowledge to solve science problems. Some studies promote students' interactions in museums (Plessis and Webb, 2008; Wishart and Triggs, 2010), in extracurricular courses with the participation of parents (Hong et al., 2013), in on-line communities of science experts or in a university setting (Smith et al., 2009).
3. **Student-centered activities: hands-on and minds-on activities**. Student-centered activities are designed to promote the students' skills and knowledge needed to solve a scientific challenge. The articles selected contain three types of activities: (a) *hands-on* activities in which students are engaged actively in practical experiences (e.g., assembling a robot); (b) *minds-on* activities in which students generate conceptual artifacts as products of their working ideas and theories (e.g., a concept map); and (c) *attitudes-on* activities in which students have to self-direct their learning (e.g., designing a plan).
  These three types of activities engaged students in sustained work focusing on the creation of shared objects of inquiry through a whole series of devices and platforms that support and allow collaborative knowledge creations. Consequently, the object-orientation of the students' activities resulted in a two-fold challenge, i.e., as a collaborative joint venture in their learning process and as a solution to a science problem. In the papers selected, the students' learning outcomes revolved around one of the following three shared-knowledge objects (see Figure 3):
  - *Construct or design of a new product*. Seitamaa-Hakkarainen et al. (2010), Lee et al. (2013), or Kim et al. (2015) engaged their students in the design of a new object by getting them to make a decision on such features as structure, materials, and function. In the same line, Sanabria and Arámburo-Lizárraga (2017) asked their students to design a digital creation using augmented reality tools. In fact, robotics has been the main technology used for constructing or designing new products (Figure 3). The ease to introduce choices, modifications, and ideas immediately has confirmed robotics as a very affordable technology for this kind of collaborative online activity. For instance, Hong et al. (2013) proposed their students to create a new robot with an eye to winning a nationwide contest. Sullivan (2011) and Ramírez-Benavides and Guerrero (2015) managed to develop a creative solution to solve a robotics problem and students ended up assembling a new robot. In a similar way, Lee et al. (2014) proposed pre-service teachers to solve technological challenges using Lego Education toolsets.
  - *Research about a scientific real-life phenomenon and production of a creative and visual communication for presentation in front of an audience*. A group of studies shared technological tools to facilitate collaborative knowledge building. These studies enhance the necessity of recognizing technology not as “playing with gadgets,” but rather as “engaging in inquiry.” From this perspective, technology becomes a collaborative environment that allows participants to follow a shared inquiry. Accordingly, if networked learning environments are used adequately, they tend to move the students' own ideas into the center rather than the periphery of discussion (Paavola and Hakkarainen, 2005). In this line of argument, Jang (2009) proposes on-line research on real-life topics as nutrition, animal evolution or diversities of organisms as a way to raise the students' curiosity and foster discussion and creativity. As an outcome of this scientific research, students elaborate a powerpoint presentation with the key concepts researched. Plessis and Webb (2008) and Wishart and Triggs (2010) propose students to use the information collected in a museum to design short interactive multimedia presentations by means of collaborative authoring tools and mobile technology.
  - *Construct a creative knowledge-object about a science topic*. Interactive digital platforms have been the most frequent devices in the studies analyzed when performing this kind of activity (see Figure 3) probably to facilitate ease of sharing, building and reflecting on ideas in a visual way. For instance, Zhang and Sun (2011) use Knowledge Forum to ask students to create new knowledge collaboratively on a science topic through reading key scientific papers. Students posed as scientists producing new knowledge through a deep understanding and interpretation of different sources. Poce et al. (2017) asked their students to write a critical and creative science text through a collaborative online platform. Using this interactive setting, students analyzed narrative scientific texts according to the elements of narratology, scientific language, and creativity and they could participate in the writing process of creative scientific texts. Similarly, Lin et al. (2016) report on how students elaborated conceptual maps collaboratively using interactive digital platforms. On the other hand, Mudaly et al. (2015) introduced the use of other technologies such as video and digital animation for performing also conceptual maps on the new roles of digital technologies for teaching purposes.
4. **Teacher's role**. Teachers play an important role in encouraging the students' activity during the learning process by promoting their students' self-directed learning and using group-based discussions to articulate, reflect upon and modify their own understanding. In the reviewed studies, the teacher creates learning opportunities to enhance students' interaction, collaboration, and knowledge creation when s/he assumed one/s of the following four roles:
  - *Enriching and organizing students' collaborative inquiry*. Teachers are in charge of guiding the phases, steps, and implementation of an advanced inquiry process. Additionally, teachers divide the inquiry process into sub-tasks with tangible sub-goals that help pave the way for the group's progress and success in achieving higher levels of creative inquiry. In this respect, teachers organize the key steps of the science inquiry by: posing questions or challenges; searching for data and evidence; generating explanations and solutions to a challenge/questions (Hemling et al., 2014).
  - *Giving specific and planned guidance*. Teachers scaffold their students' creations or elaboration of their shared ideas and thoughts by providing specific resources, templates, hints, and tools from networked databases. By offering these scaffolds, teachers help students acquire specific strategies to reach further elaboration and articulation of their own ideas and construct a better creative solution to the scientific challenge (e.g., Jang, 2009; Kim et al., 2015).
  - *Organizing the shared knowledge practices*, instead of acting as a controller of the students' learning processes. In some studies, the teacher organizes and plans the group's workflow by providing the necessary resources to help students develop intersubjectivity and commitment to negotiate their perspectives; to establish the group's ground rules for thinking together; and to enrich students' own ideas to better solve creatively the scientific challenge (e.g., Seitamaa-Hakkarainen et al., 2010; Mudaly et al., 2015).
  - *Supporting and encouraging dialogue among students to create the shared object*. Recent researchers have claimed that the students' ability to use talk collectively is crucial for collaborative creation. Thus, partners are engaged in generating a continuing and dynamic framework for their talk about their joint endeavor. Teachers can help use language purposefully for thinking, discussing and creating together, which includes aspects such as: questions, reasons, justifications, examples, and explorations. Teachers' talk is intended to help students understand that learning is an interactive process and that understanding has to be built up as a joint activity between teacher and students and among students in collaboration. By so doing, the students develop a gradual sense of responsibility for what and how they learn. Besides, this type of interaction can help students realize that knowledge is not only transmitted but also negotiated and re-created (Alexander, 2017). For instance, Seitamaa-Hakkarainen et al. (2010) conclude that the teacher's role can change the classroom culture. In this study, moving from a *transmission* culture to a *creative and collaborative working classroom* culture means that each participant is respected and his or her own voice is heard and valued to pursue a collective learning objective. Similarly, Sullivan (2011) emphasizes the importance of dialogue as a key pedagogical variable to explain the students' creative solution to a robotics problem. In this study, teachers supply a technological environment that allows students to jointly develop a shared understanding achieved through tool-mediated, communicative, and cognitive interaction.

All these teacher strategies have furnished students with the necessary creative mindset skills to solve the educational tasks proposed. Research in education has already shown that higher students' success in solving a creative task has an impact on their self-confidence and self-esteem which can be transferred to other class situations (Intasao and Hao, 2018).

## Conclusions

Collaborative creativity skills are in great demand in the current global and digital knowledge society and should be implemented taking into account all students in order to give them all the opportunity to play an active role. This paper reviews studies of designs of technology-enhanced learning environments that promote collaborative creativity skills in science education. The ultimate aim of this review has been to obtain valuable pedagogical knowledge for designing future science-related learning projects and developing future research capable of cultivating collaborative creativity using technology in the education of science.

Our work aims to fill a gap in educational research as very little research is done on collaborative creativity skills in science education. In fact, only 23 blind peer-reviewed studies met our selection criteria, i.e., which included four essential research variables to promote creativity in a global knowledge society: collaboration, creativity, technology, and science.

**Figure 4:**
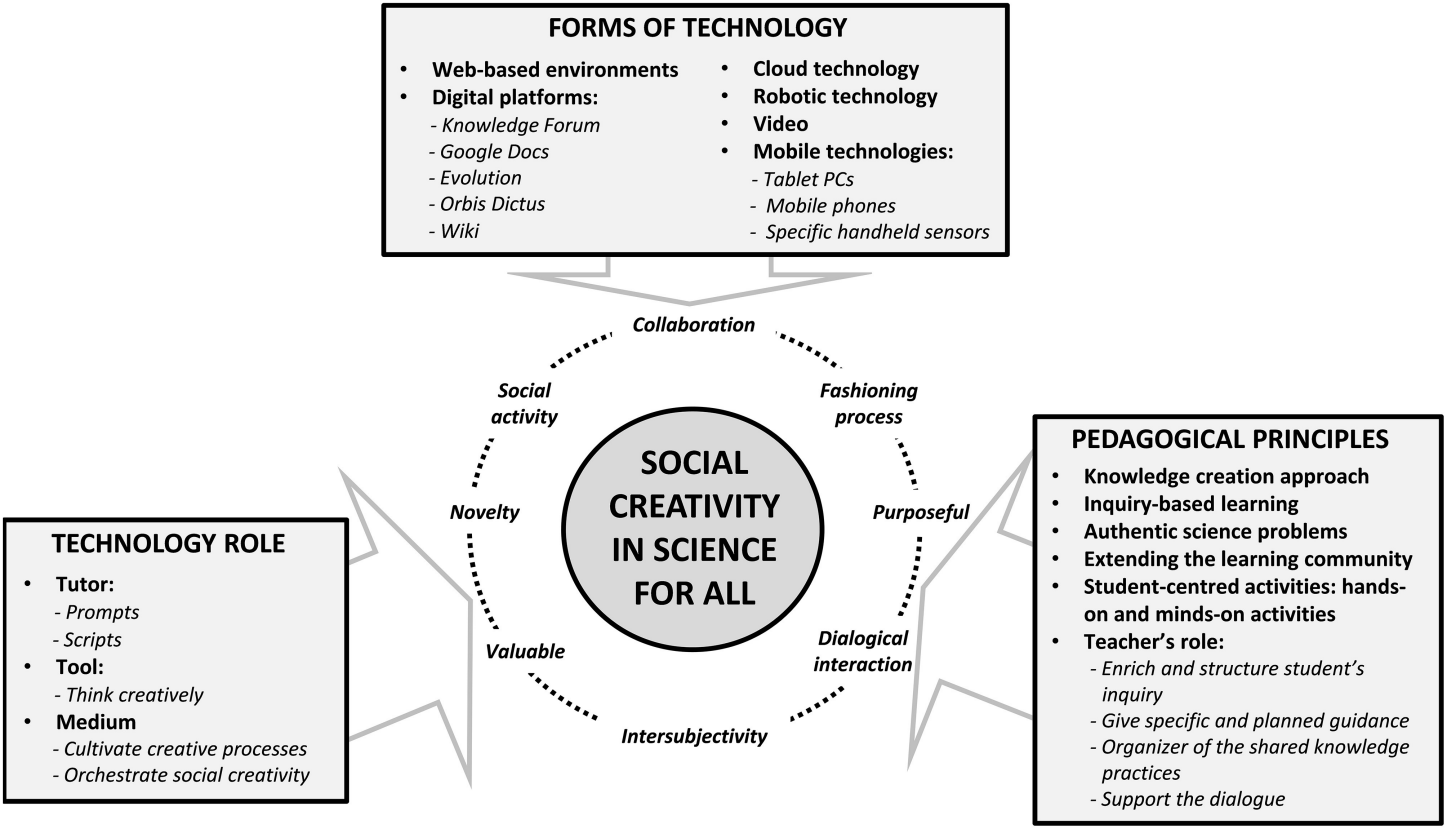
Summary about the pedagogical and technological aspects found in the reviewed studies needed to cultivate social creativity in science education.

From our study we conclude, firstly, that the design of powerful and increasingly prevailing web 2.0 technologies opens up opportunities for learners to generate, modify and evaluate new ideas through on-line and multimodal interaction. Their use can thereby support rich new forms of knowledge creation in the domain of science. Figure 4 summarizes the results of our review analysis from which we can outline three ways of promoting social creative thinking using technology (RQ 1): (1) technology as a tutor that nurtures key creative processes in science; (2) technology as a tool that shapes the students' creative thinking; and (3) technology as a resource that supplies the supportive environment to perform collective creative processes. Therefore, in our study the use of technology plays a crucial role in promoting creativity for all students.

Secondly, the reviewed studies have used several strands of technology to perform these roles. After analysing these studies, we have identified five platforms of web 2.0 technology in science education (RQ 2) teaching. They are the following: web-based environments, digital platforms, mobile technology, cloud computing technology and robotic technology. These platforms promote online learning communities that engage students in active and rich-user experience for collective knowledge creation. In all the studies analyzed, these platforms facilitated the creation of a diverse, rich and guided learning environment capable of overcoming the students' differences and learning obstacles.

Thirdly, a pedagogical approach based on knowledge creation arises as a framework capable of promoting technology-enhanced collaborative creativity in science education for all students. After analysing these studies, we identified four pedagogical advantages in the design of science knowledge creation learning scenarios (RQ 3): (1) designing authentic science problems; (2) extending the learning community outside the classroom walls; (3) designing student-centered activities that include hands-on and minds-on tasks; and (4) verifying the central role of the pedagogical uses of technology in promoting rich, new, and multimodal forms of students' learning processes. Additionally, our research study has identified four main teaching roles when promoting collaborative creativity processes with technology: organization, enrichment, orchestration, and support of the students' collaborative creativity processes with technology.

Finally, it should be noted that, despite following a methodology based on analysis of literature review and search through popular and widely-recognized databases in education (Scopus and Web of Science), there have been limitations in the process of selecting the papers, which may have influenced our search. Among these limitations are the following three: (i) only peer-reviewed papers were selected; (ii) only publications in English were considered while other publications, even key studies, in other languages were discarded; and (iii) we might have failed in developing an appropriate search terms strategy. Moreover, the broad focus of the present study (i.e., it focuses on a wide range of technologies, different educational contexts, etc.) has resulted in a heterogeneous screening of studies. The diverse nature of the studies analyzed and the fact that only 23 publications have been analyzed may have limited the impact of our conclusions and recommendations.

In a nutshell, this paper has reviewed a series of key studies in the context of science education designed to promote three highly significant educational variables for solving twenty-first century challenges: i.e., collaboration, creativity, and technology. In particular, it reveals the multiple roles and possibilities that new technology tools can offer in fostering social creativity. Our work has also revealed that both technology and pedagogy are equally important and needed to promote social creativity in science classrooms. In this respect, the teacher's pedagogical stance and understanding on how best to exploit the affordances of digital technologies are critical in determining the productive use of these tools in teaching and learning (Mercer et al., 2017, p. 9). Our paper may well-contribute to supplying equal education for all students by bringing inspiration to teachers on how to design technology-enhanced learning environments that promote collaborative creativity in science education.

## Author Contributions

All authors listed have made a substantial, direct and intellectual contribution to the work, and approved it for publication.

### Conflict of Interest Statement

The authors declare that the research was conducted in the absence of any commercial or financial relationships that could be construed as a potential conflict of interest. The handling editor declared a shared affiliation, though no other collaboration, with the authors.
